# The correlation of factors affecting the endometrial thickness with pregnancy outcome in the IUI cycles 

**Published:** 2011

**Authors:** Victoria Habibzadeh, Sayed Noureddin Nematolahi Mahani, Hadiss Kamyab

**Affiliations:** 1 Afzalipour Clinical Center for Infertility, Kerman University of Medical Sciences, Afzalipour Hospital, Kerman, Iran.; 2 Department of Anatomy and Embryology, Faculty of Medicine, Kerman University of Medical Sciences.

**Keywords:** * Endometrial thickness*, *Affecting factors*, *Intrauterine insemination*, *Pregnancy rate*

## Abstract

**Background:** Many studies have been carried out to understand the effect of endometrial thickness on the reproductive outcome while the factors affecting the pattern itself are still unknown.

**Objective**: To determine the factors such as age and the number of follicles that could affect the endometrial thickness

**Materials and Methods:** This study was conducted as a retrospective study on 680 infertile women considered for intrauterine insemination (IUI). IUI protocol was sequential regimen of clomid and gonadotropin. Endometrial thickness measurement was done on the day of HCG administration. Correlation between endometrial thickness and factors such as age, total follicle numbers, dominant follicle numbers, gonadotropine ampule numbers and pregnancy rate were assessed.

**Results:** The mean endometrial thickness was 7.2±1.8 mm. The endometrium was thinner in older patients compared with younger ones. But in all age ranges pregnancy rate was higher in endometrial thickness 6< ET≤10 mm (p<0.05).

**Conclusion:** We did not find any correlation between age, number of follicles and gonadotropine ampoules with endometrial thickness but in all age ranges, there is a possibility of higher chance of pregnancy in endometrial thickness 6 < ET≤10 mm.

## Introduction

During ovulatory cycles, pattern and thickness of endometrial is variable. After menstruation, endometrium is thin and becomes thicker gradually. Although many studies were done about affecting factors on endometrial thickness in infertile women, over the years, but the results is still unclear (1, 2). Researches suggest effect of age, etiology of infertility and factors such as dominant follicle number on endometrial thickness and some of studies suggest strong effect of endometrial thickness on the pregnancy rate (3-5). A thin endometrium is not an adequate reason for cancellation of IVF cycles (4, 6).

Some factors such as women age, infertility etiology, drug protocol, estradiol levels, previous injuries to endometrium, were supposed as important affecting factors with endometrial growth (7-9). 

Aging process in uterus is not as same as ovaries and endometrium growth is normal in response to estrogen in perimenopausal years. In some older women, myomatous uterus or PID can affect IVF results. Women age is an indirect factor on endometrium and major factor is ovaries and estrogen levels. Previous injuries to endometrium are a known factor on poor endometrium. (10, 11).

Some references explained a special limitation for appropriate endometrial thickness for pregnancy (12). In ART cycles often we see a thin endometrium with few dominant follicles or a thick endometrium with one or two small follicle. The aim of this retrospective study was to determine the effect of some factors such as age, number of follicles and number of gonadotropin ampulse on the endometrial thickness and its impact on pregnancy in intrauterine insemination cycles. 

## Materials and methods

This retrospective study was performed in Afzalipour, University Hospital of Kerman, from March 2003 to March 2007. From 720 infertile women, 680 patients (intrauterine insemination cases) were enrolled in to this study successively. 

Inclusion criteria were: anovulutory or oligovulatory women without any other infertility problems, normal sperm analysis, and at least two years infertility period. Tubal patency was assessed by laparoscopy or hystrosalpingography. We excluded mild male factor, uterine factor, endometrial and tubal factor, uterine adhesions and poor obstetric history from study. 

Some of women had a history of pregnancy (secondary infertility), and some were primarily infertile. In some studies, infertility period is an important factor and we excluded women with infertility period more than 6 years. IUI results in women older than 40 years is poor but we did IUI in 20 women > 40 years because marriage period of them was < 6 months. Our parameters, age of women, follicle numbers, gonadotropine ampoules numbers, endometrial thickness and pregnancy occurring were entered into a questionnaire. Induction ovulation protocol was clomid on 3rd day of cycle, (Iran Hormon) 100mg for 5 days and subsequently gonadotropin (75-150 unit) for 3 days. Vaginal ultrasonography was done on 11^th^ day of cycles, for determining of number and size of developing follicles.

When the mean diameter of the lead follicle reached 16-18mm, HCG, (LG life IVF-c-Korea) 10000 units was administered to trigger ovum release and intrauterine insemination was done 36-38 hours later. Endometrial thickness was measured by transvaginal ultrasonography on the day of HCC administration. Measurements were conducted in the mid sagittal plane, from the outer edge of the endometrial-myometrial interface to the outer edge of the widest part of the endometrium. Then, patients were followed for pregnancy with β-HCC testing. In hyperstimulated ovaries (total follicles) ≥15 or poor responders (no dominant follicle) we canceled cycles. In patients with >4 dominant follicles for prevention of OHSS 1 CC Buserelin was injected. After obtaining written consent, IUI cycle was done. 

**Statistical analysis**

Data was analyzed with SPSS software, to determine correlations between endometrial thickness and patients parameters. Logistic regression analysis was performed too.

## Results

In this retrospective study, from the total of 720 women, 40 patients were renounced treatment and we performed IUI cycles for 680 patients. Data were analyzed with SPSS software and statistical methods. The means age of patients was 28.5±5.3 years and 89% of them were ≤≤35 years of age (Table I). The mean endometrial thickness was 7.2±1.8mm (range, 4-18 mm). 

In 36.2% of patients endometrial thickness (ET) was ≤6 mm and in 59.4% it was 6< ET≤10 mm and in 4.4% it was >10 mm (Table I). Pregnancy rate was 12.5%. Table I presents pregnancy rates according to ET and patients age. Pregnancy rate in patients younger than 35 years old with ET≤6 mm was 8% and with 6<ET≤10 mm was 16.2%, this difference was statistically significant (p<0.05). Pregnancy rate in patients older than 35 years old with endometrial thickness ≤6 mm was 3.1% and with 6<ET≤10 mm was 10%, (p<0.05). Pregnancy rate in all age ranges with ET≤6 mm and 6<ET≤10 mm was 8.9% and 15.6% respectively (p<0.05). Pregnancy rate in all age ranges in ET>10 mm was zero. Effects of some factors such as age, follicle number, and gonadotropin ampoules numbers on ET were studied. In older patients, mean ET was lower (Table II). For example, mean ET in age range of 17-25 years was 7.4±1.98 mm and in age range of >40 years was 6.9±1.7 mm (p=0.227). With increasing the number of 16-18 mm follicles (≥6) endometrium was thicker. With increasing number of gonadotropine ampoules, endometrium was thicker but no statistical significance was considered too. In all age ranges, there was not statistical significant correlation between age, gonadotropine ampoules number and follicle numbers with ET (Table II). But there was a correlation between age, and follicle and gonadotropin ampoules numbers and ET (p< 0.05). A logistic regression analysis executed for parameters that correlated with ET≤6 mm. We found that 36-40 years old patients had an OR of 2.85 for developing a thin endometrium, compared to 17-25 years old patients, with an OR of 0.935. There was a meaningfull p-value for odd ratios, in all of parameters. 

**Table I:** Correlation between pregnancy rate with age and ET

**p-value**	**ET>10 mm**	**6 < ET ≤ 10** ** mm**	**1** ≤** ≥ET ≥** ≤ **6 mm**	**Total** **(n)**	**Pregnancy/age (years)**
	**n (%)**	**n (%)**	**n (%)**		
>0.05	27 (4.7%)	364 (60%)	214 (35.3%)	605	≤ ≤35
	-	59 (16.2%)	21 (9.8%)	80	Pregnancy + age ≤35
>0.05	3 (0.4%)	40 (53.3%)	32 (42.6%)	75	<35
	-	4 (10%)	1 (3.1%)	5	Pregnancy + age > 35
>0.05	30 (4.4%)	404 (59.4%)	246 (36.3%)	680	All age ranges
	-	63 (74%)	22 (26%)	85	Pregnancy in all ages

**Table II:** Affecting factors on ET

P-value	Mean ET(mm)	ET>10	6≤ET≤6mm	1≤ET≤6mm	Total (n)	Parameter
		N(%)	N(%)	N(%)		
0.227	7.4±1.98 7.2±1.8 6.8±1.7 6.9±1.7	14(6.4%) 13(3.4%) 2(3.6%) 1(5%)	128(58.4%) 236(16.7%) 28(50.9%) 12(60%)	77 (35.2%) 137 (35.6%) 25 (45.5%) 7 (35%)	219 386 55 20	Year 17-25 26-35 36-40 <40
01.645	7±1.6 7.1±1.8 7.2±1.7 7.3±1.8	2(3.5%) 8(3.2%) 13(6.8%) 7(3.9%)	32(56.1%) 153(60.5%) 108(56.3%) 111(62.4%)	23(40.4%) 92(36.4%) 71(37%) 60(33.7%)	57 253 192 178	Follicle number (16-18mm) 1 2-3 4-5 <6
0.252	1.8 ± 7.1 7.1 ± 1.8	6(1.1%) 24(4.5%)	78(14.7%) 326(61.4%)	65(12.2%) 181(34.1%)	149 531	HMG Amp N ≤6 <6
	7.2±1.8	30(4.4%)	4.4(59.4%)	246(36.2%)	680	Total

**Table III:** Logistic regression model for ET ≤6 mm

**p-value**	**95% confidence Interval**	**OR**	**Parameters **
			Year
0.915	0.274 – 3.19	0.935	17-25
0.364	0.665 – 3.04	1.422	26-35
0.998	-	2.85	36-40
-	-	-	40
			HMG Amp. N
0.481	0.372-8.156	1.74	≤ 6
0.390	0.673-758	1.363	6>

## Discussion

Correlation between ET and pattern with pregnancy rate and predisposing factors for growth of endometrium are unclear. In IVF cycles, we encounter the failure of pregnancy with thick endometrium and successful pregnancy with thin endometrium, despite transfer of good quality embryos (1). In this study, we evaluated endometrial thickness within 3 ranges of ≤6, 6<ET≤10 and >10 mm. We did not find correlation between age, number of gonadotropin ampule and number of follicles. Thus endometrial thickness is independent of these factors. In our research, pregnancy rate was studied, in age ranges (35> and 35< years) with different endometrial thickness (≤6, 6<ET≤10 and >10 mm). In all age ranges, pregnancy rate was lower with ET≤6 mm. Gentry studied on use of endometrial measurement as an exclusion criterion for IVF using clomiphen citrate (CC) and concluded that for CC-IVF, endometrial measurements should not be used as an exclusion criterion because still pregnancies occurred at comparable frequencies in all the groups (13).

Reuter *et al* concluded that endometrial thickness of at least 8 mm, with a high number of follicles (up to three) with an average of 15 mm are correlated with a higher rate of conception (14). This finding is similar with our data.

Tomlinson studied prognostic indicators for IUI and their logistic regression identified four significant IUI variables of follicle numbers, ET, donation of infertility and progressive motility of sperm which are the most predictive of IUI success (15). 

Hock analyzed the endometrium sonographically in patients undergoing controlled ovarian stimulation with clomiphen citrate in addition to menotropin and determined that in these patients a homogenous endometrial pattern on the day of HCG administration predicts a significantly decreased pregnancy rates compared with a trilaminar pattern (16).Tsai et al studied the role of ET and pattern and vascular impedance on IUI. They concluded that a trilaminar endometrium on the day of IUI provides a favorable prediction of pregnancy and ET and Doppler observation of the spiral and uterine arteries and dominant follicle do not have useful prediction value in CoH + IUI (17).

In our study ET was a favorable factor for pregnancy too. In Hsieh study, higher pregnancy rate and better endometrial pattern were achieved in patients with thin endometrium after aspirin administration. Aspirin therapy could not significantly increase the ET and resistance of uterine and ovarian flow (18). In Zollner study, impact of three–dimensionally measured endometrial volume on the pregnancy rates after IUI was searched and the results indicated that an endometrial volume <2 ml at the day of insemination is associated with poor likelihood of pregnancy. They advised, endometrial volume measured by 3D ultrasound is a new objective parameter to predict endometrial receptivity (19). Kolibianakis showed that endometrial thickness cannot predict ongoing pregnancy achievement in IUI cycles stimulated with clomiphen citrate (20). In Unfer study, thicker endometrium with CC and phytoestrogens was contributed to higher pregnancy rates (21).

In Esmailzadeh study, endometrial thickness on the day of HCC administration was significantly greater in cycles where pregnancy was achieved (10.1±3 vs. 7.7±3.5). The woman's age was negatively associated with pregnancy outcome, while ET and the total motile sperm count were positively associated with pregnancy outcome (22).

In Merviel study endometrial thickness of >7 mm (with triple line development) was a predictive factor for pregnancy that is similar with our study (23).

Dickey et al. studied on relationship of ET and pattern to fecundity in ovulation induction cycles and concluded that no pregnancy occurred when thickness was <6 mm, the continuing pregnancy rate was 12.6 % when thickness was ≥ 9 mm (24). 

Weismann et al study observed a detrimental effect of increased endometrial thickness >14 mm on pregnancy rates (12).

In Wiser Amir study an aging of endometrium with a negative effect of this thin endometrium only in patients >35 years of age was observed (25).

In our study in older women >35 years endometrium was thinner, (no statistically significance) but in all age ranges with suitable endomoterium pregnancy rate was greater. 

Age and uterus relation is unclear. With aging of women uterus is affected less than ovaries and it seems that with adequate estrogen levels endometrium will be thicker. 

## Conclusion

We did not find a correlation between age, number of follicles and gonadotropine ampoules with endometrial thickness but in all age ranges, chance of pregnancy is higher with endometrial thickness of 6<ET≤10 mm. 
